# Numerical Simulations of Combined Dielectrophoresis and Alternating Current Electrothermal Flow for High-Efficient Separation of (Bio)Microparticles

**DOI:** 10.3390/mi15030345

**Published:** 2024-02-29

**Authors:** Hao Jiang, Yalin Li, Fei Du, Zhaoguang Nie, Gang Wei, Yan Wang, Xiaomin Liu

**Affiliations:** 1 College of Chemistry and Chemical Engineering, Qingdao University, Qingdao 266071, China; jh13051000026@163.com (H.J.); liyalinzl0919@163.com (Y.L.); nzgqdu@163.com (Z.N.); weigroup@qdu.edu.cn (G.W.); 2 Institute of Water Chemistry, Technische Universität Dresden, D 01062 Dresden, Germany; fei.du@tu-dresden.de

**Keywords:** dielectrophoresis (DEP), alternating current electrothermal (ACET), numerical simulation, floating electrode, Joule heating, bio- and non-bioparticle separation

## Abstract

High-efficient separation of (bio)microparticles has important applications in chemical analysis, environmental monitoring, drug screening, and disease diagnosis and treatment. As a label-free and high-precision separation scheme, dielectrophoresis (DEP) has become a research hotspot in microparticle separation, especially for biological cells. When processing cells with DEP, relatively high electric conductivities of suspending media are sometimes required to maintain the biological activities of the biosample, which results in high temperature rises within the system caused by Joule heating. The induced temperature gradient generates a localized alternating current electrothermal (ACET) flow disturbance, which seriously impacts the DEP manipulation of cells. Based on this, we propose a novel design of the (bio)microparticle separator by combining DEP with ACET flow to intensify the separation process. A coupling model that incorporates electric, fluid flow, and temperature fields as well as particle tracking is established to predict (bio)microparticle trajectories within the separator. Numerical simulations reveal that both ACET flow and DEP motion act in the same plane but in different directions to achieve high-precision separation between particles. This work provides new design ideas for solving the very tricky Joule heating interference in the DEP separation process, which paves the way for further improving the throughput of the DEP-based (bio)microparticle separation system.

## 1. Introduction

Most of the cells and biological macromolecules, colloids, and polymer particles exist in the form of mixed states in nature and industrial production. The effective separation of these mixed particles has an urgent need in the fields of chemical analysis, environmental monitoring, drug screening, and disease diagnosis and treatment [1,2,3,4,5]. Chromatography and capillary electrophoresis have become mature technologies in the field of nanoparticle separation, but there are very few reports on the separation of micron-sized particles [6]. Dielectrophoresis (DEP), as a label-free, low-cost, and high-precision method for (bio)microparticle separation, has received particular research interest in recent years [7,8]. For example, Mohammed et al. [9] achieved continuous high-resolution separation of human breast cancer cells (MDA-MB-231) from white blood cells (WBCs) through the combination of inertial microfluidics and DEP. Alan et al. [10] realized the enrichment of mouse neural stem cells by using DEP technology, which made an important contribution to the separation of microparticles.

However, in the process of microparticle separation using DEP, it is inevitable to be affected by Joule heating effect induced by the electric field applied in separation. Especially in the separation of biological particles, the suspended medium of the living environment of the biosample is mostly of high electrical conductivity, so the temperature rise will be high under identical voltage conditions, which may affect the activity of the biological sample. Joule heating will lead to a temperature gradient inside the fluid, giving rise to the undesirable alternating current electrothermal (ACET) flow that disturbs the DEP trajectory of the sample to be separated and thus reduces the efficiency and accuracy of DEP-based separation. Therefore, great efforts have been made to alleviate the impact of Joule heating in order to ensure the predominance of DEP on microparticle separation. For example, Phillip et al. [11] used high-performance silicon material as the chip substrate to enhance the heat dissipation of the device and thus reduce the Joule heating-induced thermal flow, but the effect was not significant under the high electric conductivity conditions. In addition, some researchers also tried to mitigate the effects of Joule heating by tailoring electrode design, channel structure, and other operations to reduce the driving voltage while achieving identical separation effects [12,13]. In our previous work, we [14] first introduced a cooling plate to the outer wall of the channel to take away the heat generated in a timely manner, which achieved good effects but undoubtedly complicated the manufacturing process and increased the operation cost. Then, we adopted the design of hybrid floating electrodes, which could alleviate the problem of Joule heating to a certain extent. However, we found that the induced electrothermal flow could not be completely avoided. In particular, the Joule heating effect would become more obvious as long as the suspension medium has high conductivity [15]. In fact, the control and utilization of ACET flow caused by unavoidable Joule heating can be realized through rational structural design, which provides additional propulsion for sample transportation within the microchannel.

In this work, we present the feasibility and design strategy to take advantage of the undesired ACET effect for particle separation in an inhomogeneous electric field. The tailor-designed microfluidic separator can realize continuous separation of (bio)microparticles by DEP and ACET. A coupling model that includes an electric field, fluid flow field, temperature field, and Lagrangian particle tracking is established to predict microparticle trajectories in the proposed separator. Numerical simulations are carried out to evaluate the performance of the separator by employing polystyrene (PS) microspheres as model particles and different types of cells as real bioparticles. Structural and operating parameters were studied to improve the precision and efficiency of (bio)microparticle separation.

## 2. Separator Design and Numerical Simulation

### 2.1. Design Principle

Dielectrophoresis (DEP) facilitates separation based on the differences in size and/or dielectric properties among various samples. The separation principle of the (bio)microparticle separator designed in this paper is based on the fact that both the DEP force and the ACET flow work in the same plane but in different directions. For particles of different sizes, since the magnitude of DEP force is dependent on particle size, which is proportional to the third power of particle radius, the trajectory of particles with large particle sizes will shift under the action of a strong DEP force. However, the small particles are more likely to be affected by the fluid flow and biased in a different direction under the action of ACET flow, so the separation of different-sized particles could be realized. For particles with different dielectric properties, the separation is mainly based on DEP forces of different magnitudes or directions, with the assistance of electrothermal flow, which will be elaborated in the separation of biological particles. In addition, the design of floating electrodes is adopted to expand the effective working space of the DEP force and realize the maximum use of the applied electric field. The simulation process also proves that the existence of floating electrodes can effectively prevent the particles in the channel from aggregating into chains and further improve the accuracy and efficiency of DEP separation. On the basis of the above design, this paper will explore the influence of electrothermal flow on particle trajectory in a 3D separation system and then achieve high-precision separation of sample particles based on a comprehensive consideration of the influence of DEP force on particles.

### 2.2. Separator Layout

**Figure 1:**
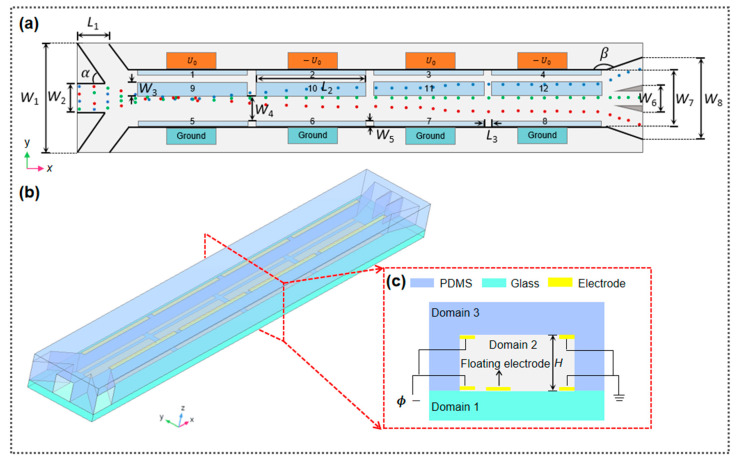
Design layout of the (bio)microparticle separator: (**a**) top view of the separator and illustration of particle separation trajectories; (**b**) 3D geometric model of the separator; and (**c**) cross-section view of the separator.

The proposed separator consists of three inlet channels (left side), a separation chamber, and three outlet channels (right side), as presented in Figure 1. For the inlet design, one sample flow channel is arranged in the middle position, and two sheath flow channels are placed on both sides with 45-degree angles (α in Figure 1a) adjacent to the sample flow inlet. The separation chamber is composed of 20 electrodes in total, with 8 driving electrode pairs (numbered from 1 to 8 in Figure 1a) orthogonally arranged on the top and bottom of the separation chamber, respectively, and 4 floating electrodes (numbered from 9 to 12 in Figure 1a) embedded in the bottom of the separation channel. The outlet channel is divided into one subchannel in the middle and two subchannels on both sides, with wedges in between for effective flow isolation. Table 1 lists the dimensions of the separator. It should be noticed that numerical analyses were conducted on all specified structural parameters of the separator to ensure optimal separation performance.

In the separation channel, the process of separating mixed samples can be broadly categorized into three phases, i.e., focusing, discrimination, and separation. The first stage refers to the microparticle indiscriminate focusing process, in which both sides of the sheath flow are adopted to enable randomly distributed particles from the sample flow to focus on lines as much as possible, allowing roughly the same initial position into the separation channel to improve the separation precision of the DEP system. During the particle discrimination stage, the bias of different directions or migration distances occurs under the combined effects of DEP and ACET flow, giving rise to distinct motion trajectories of these particles. In the third stage, particles with different sizes and/or dielectric properties can be finally collected from different outlet channels.

### 2.3. Governing Equations and Boundary Conditions

#### 2.3.1. Dielectrophoresis and ACET

Dielectrophoresis refers to the translational motion of suspended electrical particles in the dielectric medium in the presence of an external inhomogeneous electric field. When the particle’s polarizability exceeds that of the suspension medium, the particle experiences a positive DEP (pDEP) force, which drives it towards the area of maximum electric field. Conversely, when the particle’s polarizability is less than that of the surrounding medium, the particle is driven away from the region with the highest field intensity by a negative DEP (nDEP) force. For isotropic mediums with linear properties, the time-averaged DEP force acting upon a spherical particle, given a small dipole length, can be represented as follows [16]:

(1) $$F_{DEP}=2\pi a^{3}\varepsilon_{m}Re\left[K\left(\omega \right)\right]\nabla {\left|E\right|}^{2}$$

where *a* is the particle radius; $\varepsilon_{m}=\varepsilon_{r,m}\varepsilon_{0}$ is the permittivity of the medium ($\varepsilon_{r,m}$ is the relative permittivity of the suspended medium and $\varepsilon_{0}=8.854\times 10^{-12}$ F/m is the vacuum permittivity); **E** is the electric field strength and $Re[K\left(\omega \right)]$ is the real part of the Clausius–Mossotti factor. Re[K(ω)] denotes the relative polarizability of particles in the suspended medium, indicating the behavior of particles in a non-uniform electric field. For homogeneous particles like PS, $K\left(\omega \right)$ formed by the complex permittivity of suspending medium ($\varepsilon_{m}^{*}$) and particle ($\varepsilon_{p}^{*}$) can be expressed as follows:

(2) $$\;\;K\left(\omega \right)=\frac{\varepsilon_{p}^{*}-\varepsilon_{m}^{*}}{\varepsilon_{p}^{*}+{2\varepsilon }_{m}^{*}}\;\&\;\varepsilon^{*}=\varepsilon -j\sigma /\omega$$

where σ is the conductivity of the particle or suspended medium; ω is the angular frequency. For heterogeneous particles like biological cells, the application of the single-shell model enhances the accuracy of the polarization characteristics described during the simulation. Considering that the core and shell exhibit distinct dielectric properties, the $K\left(\omega \right)$ can be expressed as follows [17]:

(3) $$\;\;\;\;\;\;\;\;\;\;\;\;K\left(\omega \right)=\frac{\varepsilon_{c}^{*}-\varepsilon_{m}^{*}}{\varepsilon_{c}^{*}+2\varepsilon_{m}^{*}}$$

where the complex permittivity of the cell $\varepsilon_{c}^{*}$ can be expressed as follows:

(4) $${\;\;\;\;\varepsilon }_{c}^{*}=\varepsilon_{2}^{*}\frac{{\left(\frac{a_{2}}{a_{1}}\right)}^{3}+2(\frac{\varepsilon_{1}^{*}-\varepsilon_{2}^{*}}{\varepsilon_{1}^{*}+2\varepsilon_{2}^{*}})}{{\left(\frac{a_{2}}{a_{1}}\right)}^{3}-(\frac{\varepsilon_{1}^{*}-\varepsilon_{2}^{*}}{\varepsilon_{1}^{*}+2\varepsilon_{2}^{*}})}$$

where $a_{1}$ and $a_{2}$ respectively represent the radius of the inner membrane and the membrane-bound cell. $\varepsilon_{1}^{*}$ and $\varepsilon_{2}^{*}$ are the complex permittivity of the intracellular and membrane, respectively. When the frequencies are less than 1 MHz, Equation (4) can be expressed as follows [18]:

(5) $$Re\left[K(\omega )\right]=\frac{f^{2}-f_{0}^{2}}{f^{2}+2f_{0}^{2}}$$

where f is the electric field frequency, $f_{0}=\sigma_{m}/\pi aC_{mem}$ is the crossover frequency of the particles, and *C*_mem_ is the membrane capacitance of the cell.

The temperature gradient induced by Joule heating leads to the inhomogeneity of the dielectric properties of the fluid, which generates electrical force under the action of an electric field, resulting in ACET flow. The time-averaged electrothermal volume force giving rise to a localized vortex flow is expressed as follows [19]:

(6) $$F_{e}=\frac{1}{2}\frac{\varepsilon_{m}\left(\alpha -\beta \right)}{1+\left(\omega \varepsilon_{m}/\sigma_{m}\right)}\left(\nabla T\cdot E\right)E-\frac{1}{4}\varepsilon_{m}\alpha_{m}{\left|E\right|}^{2}\nabla T\;\;\;\;\;\;\;\;\;\;\;\;\;$$

where α and β are the thermal diffusion coefficients. For aqueous suspensions, a linear approximation is adequate: $\alpha =(\partial \varepsilon_{m}/\partial T)/\varepsilon_{m}\approx -0.04$(K^−1^), $\beta =(\partial \sigma_{m}/\partial T)/\sigma_{m}\approx$ 0.02 (K^−1^) [20].

#### 2.3.2. Governing Equations and Boundary Conditions

The physical field model used in this work for the separation of PS particles and heterogeneous cells involves the calculation of the electric field, flow field, temperature field, and particle tracking module, as well as the two coupled physical fields of electromagnetic heat and non-isothermal flow. The multiphysical field of electromagnetic heat realizes the unidirectional coupling between the electric field and the temperature field; that is, the Joule heating generated by the electric current leads to the rise of the fluid temperature, while the rise of the temperature does not in turn influence the distribution of the electric field. The non-isothermal flow multiphysical field solves the bidirectional coupling between the flow field and the temperature field. The change in fluid temperature will affect the flow pattern, and the change in flow pattern also changes the temperature distribution. The electric field is calculated based on the following Laplace’s equation [21,22]:

(7) $$\nabla^{2}\varphi =0$$

where E=−∇φ, $\;\varphi =\pm U_{0}\;$ represents the electric potential and $U_{0}$ is the voltage applied to the electrode. The calculation of flow field is based on the following Navier–Stokes and continuity equations [23]:

(8) $$\rho_{m}u_{m}\cdot \nabla u_{m}=-\nabla P+\nabla \cdot \left(\eta \nabla u_{m}\right)+F_{e}\;\;\;\;\;\;\;\;\;\;\;\;\;$$

(9) $$\nabla u_{m}=0\;\;\;\;\;\;\;\;\;\;\;\;\;\;\;\;\;\;\;\;\;\;\;\;\;\;\;\;\;\;\;\;\;\;\;\;\;\;$$

Here, $u_{m}$ is the flow rate of the medium, $\rho_{m}$ is the density of the fluid medium, P is the pressure, η is the dynamic viscosity of the fluid, and $F_{e}$ is the electrothermal force of the fluid. The energy balance equation combines electric field and thermal field to solve the temperature as follows [24]:

(10) $$k\nabla^{2}T+\frac{1}{2}\sigma_{m}E^{2}=0$$

where *k* denotes the thermal conductivity of the fluid medium, and the properties of the materials selected in this study are shown in Table 2.

**Figure 2:**
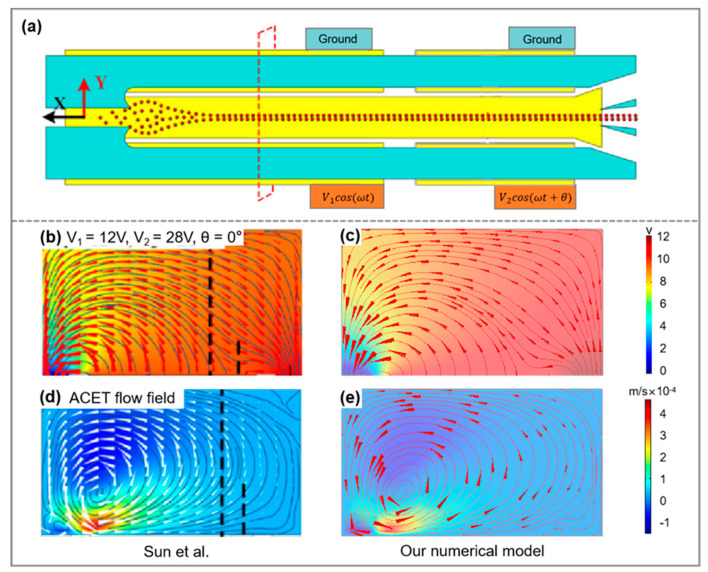
(**a**) The schematic of the separator proposed by Sun et al. [25], with the section containing the simulation results highlighted by the dashed line; (**b**,**c**) represent the electric field distributions calculated from Sun et al. and in this work, respectively; and (**d**,**e**) depict the flow field distributions calculated from Sun et al. and in this work, respectively. ($u=25\times 10^{-6}$ m/s, $\sigma_{m}\;$= 0.1 S/m, and f = 1 MHz).

In this study, a 3D computational domain is employed for the numerical simulations (Figure 2b). Detailed information on the computational domain, governing equations, and boundary conditions used for simulations is presented in Table 3. Specifically, the inlet boundary conditions for the flow field are controlled by the normal velocities of the given sample suspension ($u_{1}$) and buffer ($u_{2}$ and $u_{3}$), respectively, and the outlet is controlled by the pressure equation: P=0. The electric field distribution is mainly determined by the voltage and suspension potential applied to the electrode plate. In addition, an ideal heat dissipation condition is specified in the temperature field, with the assumption that there is a good heat exchange between the surface of the separator and the surrounding environment since the PDMS material has good heat dissipation performance. Thus, a temperature of *T*_0_ = 293.15 K is selected for the thermal boundary of the separator.

#### 2.3.3. Model and Mesh-Independence Study

The assessment of the multiphysics coupling model adopted in this study was conducted through a numerical simulation of the electric and flow fields separately, as detailed in a recently published paper. The computational domain and boundary conditions were based on those specified by Sun et al. [25]. The simulation results obtained indicate that the electric field and fluid flow state are essentially consistent with those reported in the literature (Figure 2), demonstrating the accuracy of the model used in this paper.

**Figure 3:**
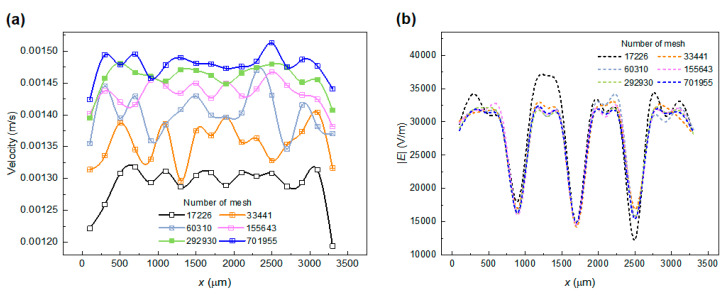
Mesh-independence analysis: (**a**) flow velocity curves and (**b**) electric field strength curves.

The mesh-independence study was carried out to ensure reliable simulation results were acquired that were independent of mesh size. Based on this, numerical simulations were performed for the sensitivity of flow field and electric field distributions on different mesh numbers (Figure 3). When the number of meshes reaches 292,930, the maximum relative errors in flow field and electric field calculations are 2.5% and 2.4%, respectively. Further increasing the mesh number has a negligible impact on the simulation results; however, it may cause an unnecessary increase in calculation expenses. Therefore, a total mesh number of 292,930 of the computational domains was adopted in the following simulation.

## 3. Results and Discussion

### 3.1. Physical Field Distribution

**Figure 4:**
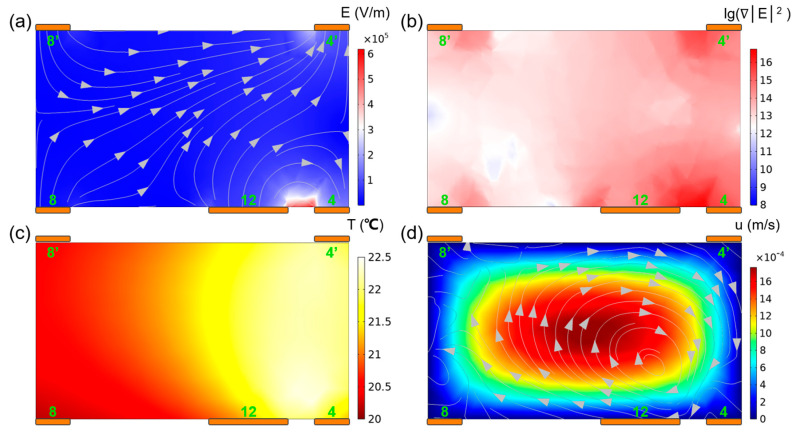
Physical field distribution diagram of the separator cross section at *x* = 2900 μm: (**a**) electric field strength; (**b**) the gradient of electric field strength squared; (**c**) temperature field; and (**d**) fluid flow velocity.

Both the electric, temperature, and flow field distributions in the separation channel were calculated, as presented in Figure 4. As expected, the maximum electric field strength and the maximum gradient of electric field strength squared appear at positions in the vicinity of the excitation electrodes, while the existence of floating electrodes expands the effective working range of DEP force. Under the influence of Joule heating, the maximum temperature rise occurs between the energizing electrode and the floating electrode. Under the uneven distribution of temperature gradient and electric field strength, a single vortex flow appears at the cross-section perpendicular to the flow direction, which can shift the particles to the side wall of the separation channel. This will provide directional guidance and a theoretical basis for subsequent particle separation.

### 3.2. Combined Effect of DEP and ACET on the Separation of PS Microparticles

To verify the design concept of the (bio)microparticle separator, standard PS microspheres with 5, 10, and 15 μm in size were employed as model particles. Moreover, the theoretical separation efficiency was adopted to evaluate the separation of PS microparticles in the separator. Here, the theoretical separation efficiency ($\eta_{i}$) of *i* particle, and the average theoretical separation efficiency ($\bar{\eta }$) of all particles are defined as the number of *i* particles ($z_{i}^{’}$) reaching the target outlet channels and the total number of *i* particles ($Z_{i}$) at three outlet channels, respectively, as follows:

(11) $$\eta_{i}=z_{i}^{’}/Z_{i}\times 100\%\;\;\;\;\;\;\;\;\;\;\;\;\;\;\;\;\;\;\;\;\;\;\;\;\;\;\;\;\;\;\;\;\;\;\;\;\;\;\;\;\;\;\;\;\;\;\;\;\;\;$$

(12) $$\bar{\eta }=\frac{\sum z_{i}^{’}}{\sum Z_{i}}\times 100\%\;\;\;\;\;\;\;\;\;\;\;\;\;\;\;\;\;\;\;\;\;\;\;\;\;\;\;\;\;\;\;\;\;\;\;\;\;\;\;\;\;\;\;\;\;\;\;\;\;\;$$

Furthermore, various structural and operation parameters, including the positions of floating electrodes, the particle initial position, the input voltage, and the inlet flow velocity, as well as the medium conductivity, were studied to explore their impact on the theoretical separation efficiency.

#### 3.2.1. Impact of the Floating Electrode Arrangement on PS Particle Separation

**Figure 5:**
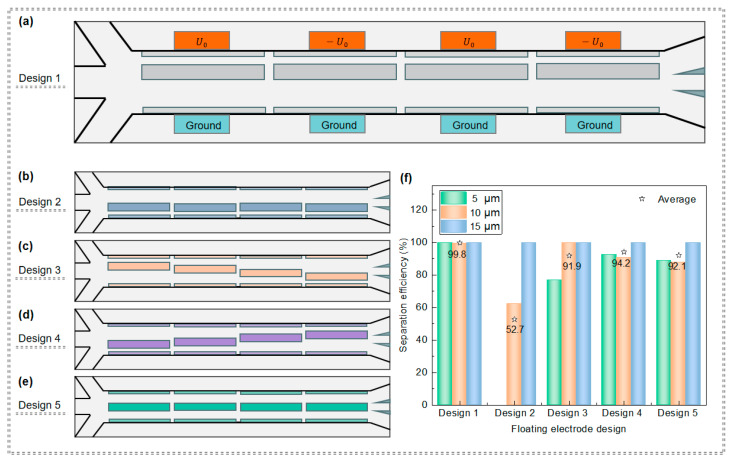
The arrangement of floating electrodes and its influence on particle separation efficiency: (**a**–**e**) different floating electrode arrangements and (**f**) separation efficiency of PS particles with different floating electrode arrangements.

The arrangement of floating electrodes has been demonstrated to pose great influences on the electric field distribution and thus affect particle motion trajectories. In this section, the theoretical separation efficiency of particles under five different floating electrode layouts (designs 1–5 in Figure 5) is mainly explored, with an optimal floating electrode arrangement suitable for particle separation being screened out, as presented in Figure 5. Among five different floating electrode designs, design 1 and design 2 are those floating electrodes arranged closed to the excitation electrode side and ground electrode side, respectively (Figure 5a,b); the floating electrodes in designs 3 and 4 are in ladder arrangements up and down, respectively (Figure 5c,d); and the floating electrodes in design 5 are embedded in the middle of the separation channel (Figure 5f). It was found that the floating electrode mainly acts in the high potential field; that is, when the floating electrode is distributed on the side of the electrode where the voltage is applied, the average theoretical separation efficiency of particles is higher, up to 99.8%. In this case, a counterclockwise vortex heat flow is formed on the cross section of the channel perpendicular to the fluid flow. The trajectory of PS particles can be biased to the side of the channel opposite to the nDEP force so as to interact with the DEP force to enable high-precision separation between particles. Therefore, design 1 with floating electrodes placed on the side of the excitation electrodes was adopted in the following simulations (Figure 5a).

#### 3.2.2. Impact of Operating Parameters on PS Particle Separation

**Figure 6:**
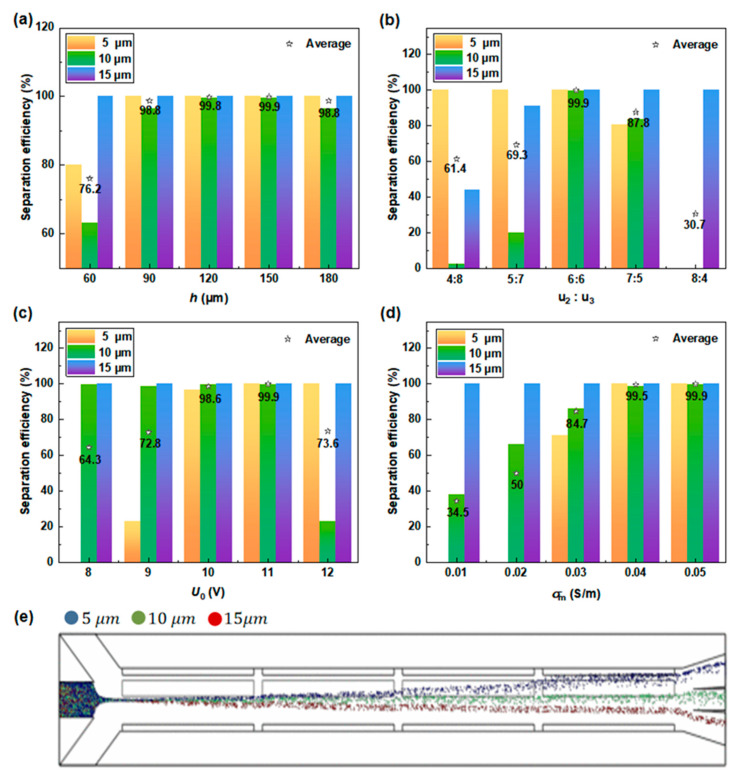
Study on single parameters affecting particle separation efficiency: separation efficiency of PS particles at (**a**) different particle inlet heights; (**b**) different sheath flow rates; (**c**) different driving voltages; (**d**) different media conductivity; and (**e**) particle trajectories with sizes of 5, 10, and 15 μm under the optimal operating parameters.

The initial injection position of particles was found to affect the final separation efficiency due to the dependence of the ACET vortex flow on particles’ lateral displacement at different heights. Therefore, the inlet height (*h*) of particles was adjusted to analyze its influence on particle separation efficiency (Figure 6a). It was found that the low entrance height of particles leads to a poor separation effect because the flow velocity at the bottom channel is very small under the non-slip velocity boundary, and thus the induced ACET vortex flow exerts little effect on particles in the form of drag force. Moreover, particles in the injection position near the channel bottom are prone to colliding with the channel wall due to the gravitational force, which may cause particle adhesion to the wall and reduce the particle recovery rate. When the initial height of particles reaches a certain position, the particle separation efficiency is no longer sensitive to the change in height. Under this operating condition, the average separation efficiency of PS particles of different sizes reaches up to 99.9% at an initial height of *h* = 150 μm. Therefore, the particle injection height of *h* = 150 μm was selected for the following parametric studies.

The velocities of the two sheath flows have obvious impacts on particles’ separation efficiency, as illustrated in Figure 6b. Low sheath flow inlet velocity at the driving electrode side ($u_{2}$) gives rise to poor separation efficiency for 10 μm particles. This is due to the strong sheath flow velocity at the grounding electrode side ($u_{3}$), which deflects the trajectory of all particles to the driving electrode side, in which small-sized particles are more susceptible to the fluid flow to produce trajectory deviations. This phenomenon is beneficial for the separation of 5 μm particles, which makes them closer to the target outlet channel, but not friendly for the separation of 10 μm particles. The effect of DEP force is strongly dependent on particle size (Equation (1)). Under the effect of nDEP force (*f* = 1 kHz), 15 μm particles can be effectively pushed to the ground electrode side to reach the target outlet channel. On the contrary, high sheath flow velocity at the driving electrode side results in poor separation for small-sized particles. Under strong sheath flow $u_{2}$, particles are all shifted to the ground electrode side. This is beneficial to the improvement of the separation efficiency of 15 μm PS particles but not conducive to the separation of 5 and 10 μm particles. The average theoretical separation efficiency of particles reaches 99.9% when the velocity of sheath flows on both sides is identical. Therefore, an identical sheath flow velocity of ${\;u}_{2}$=${\;u}_{3}$= $6\times 10^{-4}$ m/s is selected for the PS particle separation studies.

The driving voltage, which is one of the key parameters determining the distribution of electric field and flow field, can affect both the DEP force and the drag force applied to particles, thus further impacting the particle trajectory and the separation efficiency (Figure 6c). The change in input voltage is equivalent to the variation of the relative proportion of the DEP force and the ACET force acting on the particles, since the induced DEP velocity increases quadratically with the electric field strength, while the ACET flow is proportional to the fourth power of the electric field strength [26]. With the decrease in the driving voltage, the ACET effect attenuates more obviously as compared to DEP, resulting in insufficient ACET flow on small-sized particles to drive them to predetermined outlet channels. On the other hand, the separation efficiency of small-sized PS particles can be improved by increasing the applied voltage due to the significantly enhanced ACET effect. Further increasing the voltage may lead to excessive ACET against DEP, where 10 μm particles cannot be completely collected from the target outlet channel. The separation effects of PS particles with three different sizes perform best under an input voltage of *U*_0_ = 11 V, with an average separation efficiency of 99.9%.

The influence of the fluid medium conductivity on the separation of PS particles is mainly reflected in the direct Joule heating effect. The uneven Joule heating leads to a change in fluid conductivity and dielectric properties, thereby forming the ACET flow and indirectly acting on the particles through drag force, thus affecting the particle trajectory. At low medium conductivity (below 0.04 S/m in Figure 6d), the insufficient Joule heating results in very weak displacement of particle trajectories to the driving electrode side, which is not conducive to small-sized particles reaching the target outlet. In the case of high medium conductivity (above 0.04 S/m in Figure 6d), the high temperature rise induced by Joule heating promotes effective discrimination among different-sized particles, giving rise to satisfactory separation efficiency.

In addition, the influence of the electric field frequency (ranging from 60 kHz to 140 kHz) on particle separation efficiency was explored, with negligible impact being observed on particles. This is because PS particles are always affected by nDEP in the investigated frequency range, so the change in frequency has little influence on their trajectories. Under the above optimal operating parameters (i.e., $u_{1}$ = $3\times 10^{-4}$ m/s, ${\;u}_{2}$ = ${\;u}_{3}$= $6\times 10^{-4}$ m/s, *U*_0_ = 11 V, $\sigma_{m}$ = 0.05 S/m, and f = 1 kHz), the trajectories of three sized PS particles are presented in Figure 6e.

### 3.3. Separation of Biological Cells

The high-efficiency separation of different-sized PS particles was successfully achieved by the proposed DEP separator. In the following section, the practical application of the proposed separator will be evaluated through numerical simulation of continuous separation of biological samples, i.e., the isolation of circulating tumor cells (CTCs) from normal blood cells, the separation of red and white blood cells, as well as the separation of viable/nonviable yeast cells.

#### 3.3.1. Isolation of CTCs from Normal Blood Cells

RBCs, granulocytes, and MDA-MB-231 cells (as the numerical model for CTCs) were used as biological particle models to examine the performance of the proposed separator [27]. To mimic the huge concentration differences between CTCs and normal blood cells in a real patient’s blood, the concentration of MDA-MB-231 (10^6^ cells/mL) was specified as three orders of magnitude lower than that of blood cells (10^9^ cells/mL) in the simulations. In this study, the numerical separation process between the three different types of cells was implemented based on two steps: the first step was to isolate MDA-MB-231 cells from normal blood cells (including RBCs and granulocytes); the second step was to separate two different types of blood cells.

**Figure 7:**
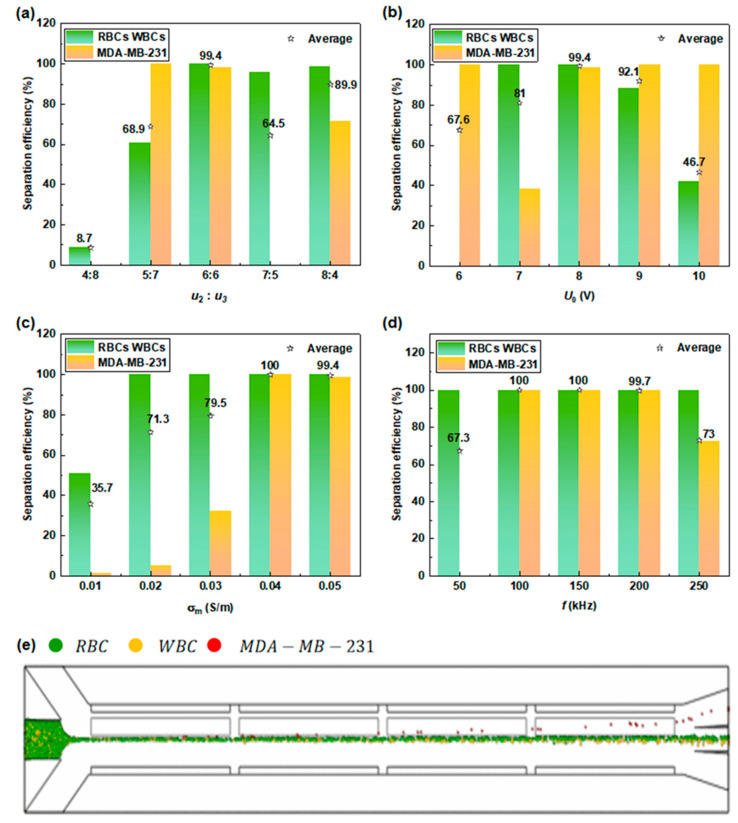
Study on operating parameters affecting the separation efficiency between normal blood cells and CTCs: (**a**) sheath flow rates; (**b**) driving voltages; (**c**) medium conductivities; and (**d**) electric field frequencies. (**e**) Trajectories of cells under the following optimal operating parameters: $u_{1}$= $3\times 10^{-4}$ m/s, $u_{2}$ = $u_{3}$ = $6\times 10^{-4}$ m/s, *U*_0_ = 8 V, $\sigma_{m}\;$ = 0.04 S/m, and f = 100 kHz.

The continuous separation of MDA-MB-231 cells from normal blood cells was verified using the proposed separator, while the impact of operating parameters on the theoretical separation efficiency was explored (Figure 7). Successful separation between MDA-MB-231 and normal blood cells can be achieved under optimal conditions. Note that at the chosen frequency (i.e., f= 100 kHz), blood cells are subjected to almost identical nDEP and ACET forces while in the opposite direction, such that their trajectories do not deviate too much in the transverse direction. On the contrary, obvious deflections can be found for MDA-MB-231 cells due to the favorable ACET force as well as the negligible DEP force caused by a nearly null CM factor at this frequency. Under the action of ACET, MDA-MB-231 cells were pushed toward the upper outlet channels, thereby achieving good separation between MDA-MB-231 and blood cells (Figure 7e).

Among other operating parameters, the sheath flow rate is also a key factor affecting the position of cells in the separation channel. Either a higher or lower than 1 sheath flow ratio of ${\;u}_{2}$ and ${\;u}_{3}$ gives rise to poor separation efficiency (Figure 7a). The applied voltage and the medium conductivity are closely related to DEP and ACET effects; hence, the rational selection of both parameters should be considered comprehensively. In addition, when adjusting both the applied voltage and the medium conductivity, it is not only necessary to consider the separation efficiency but also essential to ensure the physiological structure and activity of cells, avoiding dielectric breakdown and cell apoptosis induced by a high temperature gradient.

#### 3.3.2. Separation of Red and White Blood Cells

**Figure 8:**
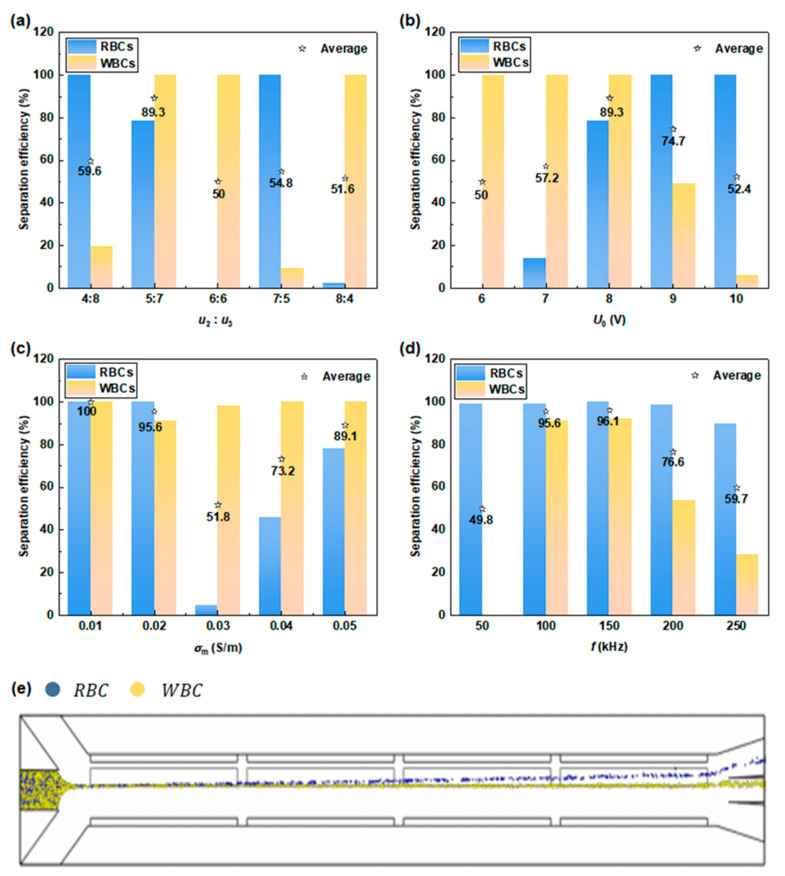
Study on single parameters affecting the separation efficiency of RBCs and WBCs. Separation efficiency of RBCs and WBCs at different (**a**) sheath flow rates, (**b**) driving voltages, (**c**) media conductivity, and (**d**) frequencies. (**e**) The trajectories of RBCs and WBCs under optimal operating parameters.

Numerical simulation was used to discover the optimal separation conditions between RBCs and granulocytes (Figure 8a–d). When the fluid conductivity is 0.02 S/m, the CM factor value of RBCs at the optimal operating frequency (f= 150 kHz) is nearly zero, thus the influence of DEP force on RBCs can be ignored. Under the effect of ACET, the trajectory of RBCs deflects to the driving electrode side, where they can be collected in the upper outlet channel. On the contrary, granulocytes show very slight deflection to the driving electrode side under the combined effects of DEP and ACET, giving rise to a large number of cells being collected by the middle outlet channel (Figure 8e). By adjusting the operating parameters, under the conditions of $u_{2}$= $5\times 10^{-4}$ m/s, $u_{3}$= $7\times 10^{-4}$ m/s, *U*_0_ = 8 V, $\sigma_{m}$= 0.02 S/m, and *f* = 150 kHz, the average separation efficiency of the two types of cells was more than 96%. Based on these results, the feasibility of separating RBCs, granulocytes, and MDA-MB-231 cells was verified (Figure 8).

#### 3.3.3. Separation of Viable and Nonviable Yeast Cells

**Figure 9:**
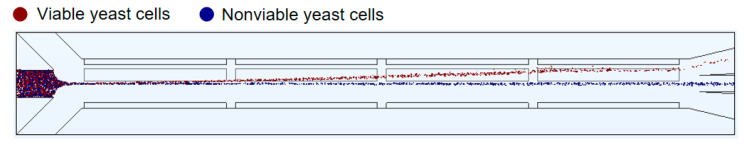
Separation trajectories of viable/nonviable yeast cells under the following optimal operating parameters: $u_{1}$ = $3\times 10^{-4}$ m/s, $u_{2}$ = $u_{3}$ = $6\times 10^{-4}$ m/s, $\sigma_{m}\;$= 0.01 S/m, and f = 1 MHz.

To evaluate the versatility of the proposed separator, the distinction between viable and nonviable yeast cells was numerically verified based on their different electric properties (see Table 4). As presented in Figure 9, viable/nonviable yeast cells are successfully separated at a fluid conductivity of 0.01 S/m and an electric frequency of 1 MHz. The CM factor of viable cells is positive, which is affected by pDEP, while the CM factor of nonviable cells is negative, which is affected by nDEP. The separation of yeast cells was realized according to the difference in force direction.

## 4. Conclusions

In summary, we reported a novel separator designed for the continuous separation of (bio)particles based on differences in size/electrical properties. The separator consists of three inlet channels and three outlet channels, along with a separation channel composed of eight pairs of excitation electrodes and four floating electrodes, capable of achieving sheath flow-assisted ternary microparticle separation. A coupling model was established that incorporates electric field, flow field, and thermal field to predict the microparticle trajectory within the proposed separator. The design of the separator lies in the full utilization of the Joule heating-induced ACET flow in combination with the DEP force synergistically acting on particles to achieve accurate separation among different particles. For the proof-of-concept study, the separation of PS microspheres based on size and the separation of blood cells based on dielectric properties were numerically achieved via the separator. By adjusting the flow rate, voltage, and other operating parameters, the efficient separation of 5, 10, and 15 μm PS particles was achieved, with an average theoretical separation efficiency of 99.9%. Moreover, the complete isolation of CTCs from blood cells was numerically realized under optimum operating conditions. Further, we demonstrated the successful separation of RBCs and WBCs with a theoretical separation efficiency of more than 96%. Finally, the successful separation of live and non-live yeast cells was confirmed through the proposed separator. Overall, this work is anticipated to provide a theoretical basis for making use of the synergistic effect of DEP and ACET, allowing high-efficient separation of sensitive biosamples.

## Figures and Tables

**Table 1:** Specification of characteristic dimensions of the separator.

Parameters	*L*_1_	*L*_2_	*L*_3_	*W*_1_	*W*_2_	*W*_3_	*W*_4_	*W*_5_	*W*_6_	*W*_7_	*W*_8_	*H*	α	β
**Value (** **μm)**	210	775	50	560	150	68	120	30	110	270	390	152	45°	166.5°

**Table 2:** Material properties used in the model [23].

	PS	RBSs	Granulocytes	MDA-MB-231	Medium
Diameter (µm)	5, 10, 15	5	9.42 ± 0.46	12.4 ± 1.16	
Density (kg/m^3^)	1050	1050	1050	1050	1000
Dynamic viscosity (Pa·S)					0.001
Conductivity (S/m)	8, 2, 1.14($\times 10^{-4})$	0.31	0.6	0.62	0.001~0.055
Dielectric constant	2.55	59	151	52	80
Membrane conductivity (S/m)		$1\times 10^{-6}$	$1\times 10^{-6}$	$1\times 10^{-6}$	
Dielectric constant of membrane		4.44	5	11.75	
Membrane thickness (nm)		9	4	4	

**Table 3:** Computation domain and boundary conditions employed in COMSOL multiphysics.

Current Module	Domain/Boundary Conditions (Scope of Application)	Governing Conditions/Equations
	Conservation of current (domains 1 and 2)	$\nabla \cdot J=Q_{J,V}J=\sigma E+J_{e}E=-\nabla V$
	Initial values (fields 1 and 2)	
	Electrical insulation (wall)	n·J=0
	Potential 1 (electrodes 1, 1’, 3, and 3’)	$U_{0}$
	Potential 2 (electrodes 2, 2’, 4, and 4’)	${-U}_{0}$
	Ground (electrodes 5, 5’–8, and 8’)	U=0
	Suspension potential (electrodes 9–12)	$\int_{\partial \Omega }^{\;}-n\cdot Jds=I_{0}$
Laminar flow module		
	Entrance (entrances 1, 2, and 3)	$u_{1},u_{2},u_{3}$
	Export (exports 1, 2, and 3)	P=0
	No slip (wall, electrode)	u=0
	Volume Force (domain 3)	$F_{e}=\frac{1}{2}\cdot \frac{\varepsilon_{m}\left(\alpha -\beta \right)}{1+\left(\omega \varepsilon_{m}/\sigma_{m}\right)}\left(\nabla T\cdot E\right)E-\frac{1}{4}\varepsilon_{m}\alpha_{m}{\left|E\right|}^{2}\nabla T$
Heat-transfer fluid		
	Entrance (entrances 1, 2, and 3)	$-n\cdot q=d_{z}\rho ∆Hu\cdot n$
	Export (exports 1, 2, and 3)	−n·q=0
	Temperature (wall)	$T_{0}=293.15\;K$
Particle tracking		
	Entrance (entrance 2)	$q=q_{0}$ $v=v_{0}$
	Export (exports 1, 2, and 3)	
	Gravity (domain 3)	$F_{g}=m_{p}g\frac{\rho_{p}-\rho_{m}}{\rho_{p}}$
	Drag (domain 3)	$F_{d}=\frac{1}{\tau_{P}}m_{P}\left(u-v\right)\tau_{P}=\frac{\rho_{P}d_{P}^{2}}{18\mu }$
	DEP force (domain 3)	$F_{DEP}=2\pi a^{3}\varepsilon_{m}Re[k\left(\omega \right)]\nabla {\left|E\right|}^{2}$

**Table 4:** Properties of viable/nonviable yeast cells used for numerical simulation [23].

Dielectric Properties	Viable Yeast Cell	Nonviable Yeast Cell
Internal radius $r_{cyto}$ (μm)	3.772	3.242
Membrane radius $r_{mem}$ (μm)	3.78	3.25
Wall radius $r_{wall}$ (μm)	4	3.5
Intracellular dielectric constant $\varepsilon_{cyto}$	50	50
Membrane permittivity $\varepsilon_{mem}$	6	6
Cell wall permittivity $\varepsilon_{wall}$	60	60
Intracellular electrical conductivity ${\;\sigma }_{cyto}$ (S/m)	0.2	7 × 10^−3^
Cell membrane conductivity $\sigma_{mem}$ (S/m)	2.5 × 10^−7^	1.6 × 10^−4^
Cell wall conductivity $\sigma_{wall}$ (S/m)	1.4 × 10^−2^	1.5 × 10^−3^

## Data Availability

Data are contained within the article.
